# Gut Entrapped in the Thorax: A Rare Presentation of Abdominal Pain in Chilaiditi Syndrome

**DOI:** 10.7759/cureus.5180

**Published:** 2019-07-20

**Authors:** Syed Hamza Bin Waqar, Osama Mohiuddin, Anosh Aslam Khan, Moiz Ehtesham

**Affiliations:** 1 Internal Medicine, Civil Hospital Karachi, Dow University of Health Sciences, Karachi, PAK; 2 Internal Medicine, Dow University of Health Sciences, Karachi, PAK; 3 Internal Medicine, Dow International Medical College, Dow University of Health Sciences, Karachi, PAK

**Keywords:** diaphragm, chilaiditi syndrome, volvulus, psuedopneumoperitoneum, acute abdomen, colon interposition

## Abstract

Chilaiditi syndrome is a rare disorder comprising of the interposition of the gut between the diaphragm and liver. This can lead to a spectrum of gastrointestinal and respiratory presentations, primarily in the elderly population in whom the disorder is relatively more prevalent. We present a case of a 63-year-old man who presented to our setup with abdominal pain and shortness of breath and later got diagnosed with Chilaiditi syndrome. He was managed conservatively and showed complete resolution of the symptoms.

## Introduction

Abdominal pain in an elderly patient can present in a variety of ways. Chilaiditi syndrome is one such presentation in which the bowel gets interposed between the liver and diaphragm, causing a range of symptoms that can easily be confused with numerous other medical diagnoses. Radiographically, it can be confused with pneumoperitoneum and thus, create challenges in medical decisions [1]. Here, we present a case of a 63-year-old elderly man who presented with abdominal pain and breathlessness. He was later diagnosed with Chilaiditi syndrome and got treated with non-surgical and conservative measures.

## Case presentation

A 63-year-old elderly man, with a known case of hypertension and chronic bronchitis, presented with progressive abdominal pain in the epigastrium and right upper quadrant for the last eight hours. The pain was continuous, moderate in intensity, dull and achy in nature. He reported no shifting or radiation of pain but did complain of constant nausea and abdominal fullness that began a few hours after the onset of pain. The patient had also vomited three times before the current presentation; vomitus was nonmucoid, non-bloody, nonbilious, non-projectile, and watery in composition with no associated retching. Post-admission, the patient also complained of being short of breath while ambulating towards his bed. He had no preceding history of altered bowel movements, fever, trauma or similar prior episodes.

On admission, the patient was fully responsive, alert, and oriented with a normal affect but appeared to be in obvious distress. He was afebrile with a pulse of 87 beats per minute (BPM), blood pressure (BP) of 135/89 mmHg, and respiratory rate of 18/min. On examination, his abdomen was marginally distended, tympanitic, mildly tender to deep palpation more pronounced in the epigastrium and right upper quadrant with normoactive bowel sounds and no hepatosplenomegaly. Pulmonary auscultation revealed bilateral audible breath sounds with bronchovesicular breathing and added rhonchi which cleared on coughing. Oxygen saturation was 97%. Neurological exam for bulk, tone, power, and reflexes was insignificant. The sensory and cerebellar system were also intact. Cardiovascular exam was unremarkable with regular rate, rhythm, and no extra heart sounds or murmurs. No cyanosis, edema, conjunctival pallor, jaundice, or clubbing were appreciated.

Appropriate lab workup was done, which included: complete blood count, liver function tests, basic metabolic panel, and random blood sugar levels. The obtained results were insignificant with normal values except for a marginally low potassium level of 3.2 mEq/L (normal: 3.5-5.0 mEq/L). Erythrocyte sedimentation rate and C-reactive protein levels were also within normal limits and so was the Coagulation profile.

**Figure 1:**
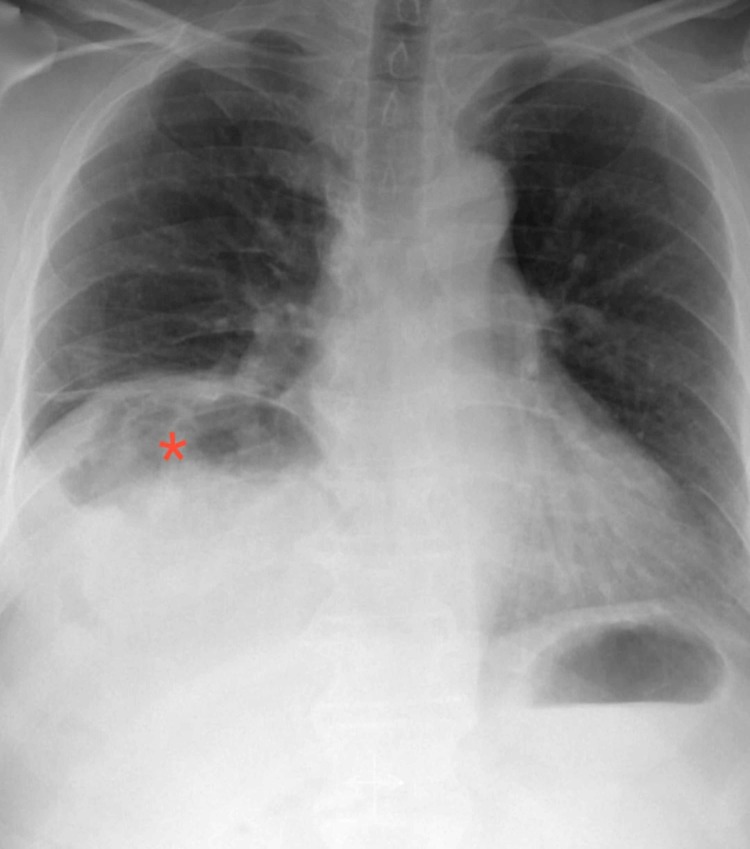
Chest X-ray showing sub-diaphragmatic gas (*) under the right hemidiaphragm

Due to the concomitant presentation of abdominal pain and shortness of breath during ambulation; basic imaging studies including chest radiograph, and abdominal ultrasound were requested. Chest radiograph revealed gas between the liver and right-sided hemidiaphragm, which raised concerns for pneumoperitoneum (Figure 1).

Ultrasound (US) abdomen showed gas echoes in hepato-diaphragmatic space; echoes did not change location despite altering the patient’s position while imaging. This was a point of concern for the primary team due to this being a rather atypical presentation for pneumoperitoneum. The patient was given nothing by mouth during the initial phase, and intravenous fluids were administered to maintain euvolemia while awaiting computed tomography.

**Figure 2:**
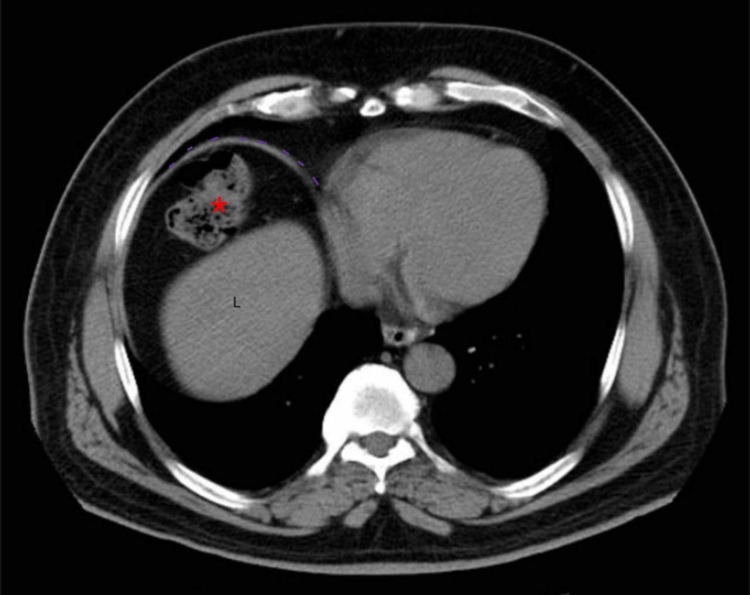
Axial non-contrast computed tomography (CT) showing interposition of the large intestine (*) in between the liver (L) and diaphragm (dotted lines)

**Figure 3:**
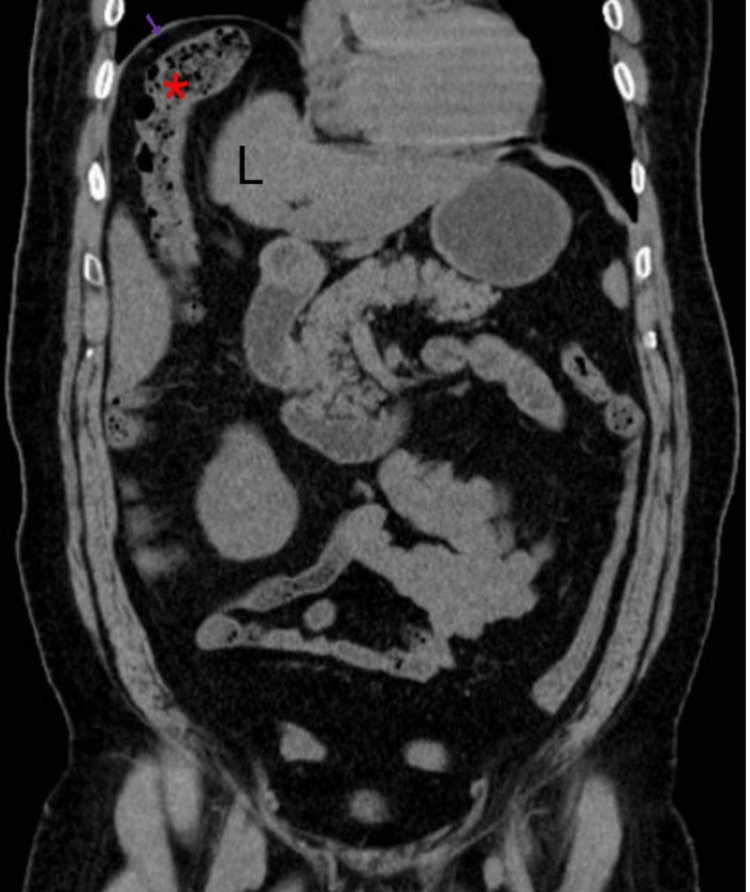
Coronal non-contrast computed tomography (CT) showing interposition of the large bowel (*) in between the liver (L) and diaphragm (shown by arrow) in accordance with Chilaiditi syndrome

Computed tomography (CT) of the abdomen was obtained to further resolve the mystery of subdiaphragmatic gas, which could either be free or intraluminal. CT displayed interposition of large intestinal loops between the diaphragm and the liver with no free intraperitoneal air or fluid. There were no signs of volvulus, ischemic colitis or perforation (Figures 2-3).

Based on these explicit findings, negating pneumoperitoneum, a diagnosis of Chilaiditi syndrome was consensed.

The patient was aborted from an initially planned laparoscopy and rerouted to conservative measures: he was initiated on metoclopramide, enemas, and laxatives. Bowel decompression was started with a nasogastric tube attached to intermittent suction and a rectal tube attached to a gravity drainage system. Post-decompression radiograph was obtained which did not reveal air under the diaphragm. The patient gradually responded to conservative measures and a weekly follow-up documented complete resolution of symptoms.

## Discussion

Chilaiditi syndrome is one of the rarest intestinal diseases, comprising of the interposition of the colon or small bowel in hepato-diaphragmatic space. It was first documented back in 1865 and it presents with a wide array of gastrointestinal symptoms [1]. Radiographically, the appearance of bowel in between the right hemidiaphragm and liver is termed as Chilaiditi sign. This sign was named after a Greek radiologist Demetrius Chilaiditi in 1910, who reported three incidental cases of free air in the abdomen on routine abdominal and chest X-rays due to this displacement. Chilaiditi sign with gastrointestinal symptoms is referred to as Chilaiditi syndrome [1-2].

Chilaiditi sign is incidentally found in 0.025% to 0.28% of chest and abdominal X-ray films and around 1.18% to 2.4% of abdominal computed tomography (CT) scans. The frequency of the sign occurring in males is four times that of females. Though the sign has been reported in the age bracket of five months to 81 years, it is more evident in aged individuals where the incident rate is around 1% [1].

Normally, suspensory ligaments of the liver, mesocolon, falciform ligament and liver itself are anatomically positioned in such a way that the area around the liver is minimized and this restricts interposition of the colon. Chilaiditi syndrome, however, can occur due to various congenital and acquired causes: congenital factors include the absence of suspensory or falciform ligaments, redundant colon, paralytic right hemidiaphragm, malposition, and dolichocolon while acquired factors include multiple pregnancies, obesity, liver pathologies such as cirrhosis and atrophy, ascites, liver ptosis caused by loosening of ligaments. Other risk factors include pathologies of intestine like megacolon, absence of peritoneal attachments, volvulus, meteorism, and colonic hypermobility. Diaphragmatic abnormalities such as phrenic nerve injury and intrathoracic pressure changes as seen in cases of chronic obstructive pulmonary disease (COPD) may also contribute to the pathogenesis of Chilaiditi syndrome [2-4].

Anatomically, Chilaiditi sign is classified as anterior or posterior, depending upon the positioning of interposed bowel with respect to the liver. Most commonly affected bowel segments are the hepatic flexure, ascending colon and transverse colon. However, a few cases also reported the involvement of small bowel, either with or without colon [1]. Clinically, Chilaiditi sign can appear as an asymptomatic anatomic abnormality while Chilaiditi syndrome can cause a range of gastrointestinal symptoms such as abdominal pain, anorexia, nausea, emesis, distention, constipation, flatulence and referred right shoulder pain due to diaphragmatic irritation. Other complications like cardiac arrhythmias, dyspnea, substernal pain, volvulus, and bowel obstruction may require emergency management [1,4-5]. Literature review reveals several conditions that are associated with Chilaiditi syndrome including COPD, congenital hypothyroidism, pneumatosis cystoides intestinalis, paralytic ileus, melanosis coli, sigmoidal or rectal tumors, scleroderma, mental retardation and psychiatric disorders [1]. Due to the rarity of this syndrome, it is essential for surgeons to undertake a thorough medical history and physical examination followed by radiographic studies for confirmation of Chilaiditi syndrome. Apart from exhibiting free air under the diaphragm, findings on plain abdominal X-ray include ileus and impaction of stool. Close inspection of left lateral decubitus abdominal X-ray film may reveal colonic haustra under the right hemidiaphragm which helps to differentiate Chilaiditi sign, also known as pseudo-pneumoperitoneum, from true pneumoperitoneum [1,6]. Despite classic findings on abdominal films, computed tomography (CT) scan is now considered the investigation of choice. Distinctive radiographic findings on CT scan include air under the diaphragm with visible haustra, absence of haustra displacement with changes in patient’s position, elevation of right hemidiaphragm and caudal displacement of the liver due to the interposing bowel segment, and depression of superior margin of liver below left hemidiaphragm. Henceforth, CT scan plays a decisive role in differentiating Chilaiditi syndrome from conditions with similar clinical features such as diaphragmatic hernia, subphrenic abscesses, and pneumoperitoneum; limiting the need for unnecessary surgeries [7].

Identification of Chilaiditi sign is considered extremely significant prior to certain procedures. Its recognition helps in preventing the risk of bowel perforation during percutaneous transhepatic interventions or liver biopsies, especially in cirrhotic patients. Studies have shown that Chilaiditi sign also complicates colonoscopies due to continuous entrapment of air in the interposed bowel segment which increases intraluminal pressure and may eventually lead to perforation. It has, however, been observed that administration of carbon dioxide as an insufflating agent reduces the risk of bowel perforation [8].

Treatment of Chilaiditi syndrome varies according to the severity of the clinical picture. Asymptomatic patients with Chilaiditi sign often do not require any intervention. Mild to moderate cases are managed conservatively which entails bed rest, nasogastric decompression, intravenous fluids, enemas, analgesia and introduction of high fiber diet and stool softeners. CT scan is routinely repeated which should correspond with clinical improvement and show resolution of the interposition. Surgical management is reserved for patients who do not respond to conservative measures or develop complications like perforation, bowel ischemia, cecal or colonic volvulus, subphrenic appendicitis, and internal herniation. Surgical interventions range from fixation of the gut to colonic resection; depending upon the length and condition of the involved bowel segment. Colopexy is recommended for uncomplicated cecal volvulus while resection is mandated for gangrene and perforation. However, colonic resection is the best intervention for resolving colonic volvulus. Since colonic volvulus has about 16% risk of gangrene development, colonoscopic reduction is not recommended [1-3,7].

## Conclusions

Chilaiditi syndrome is an unusual manifestation of intestinal obstruction caused by the interposition of the gut between the liver and diaphragm. Abdominal pain with atypical findings of subdiaphragmatic gas in Chilaiditi syndrome can lead to incorrect and drastic decisions in the treatment of the patient. Hence, it is crucial for physicians dealing with the geriatric population to be vigilant of such findings and utilize CT scan to reach the diagnosis before proceeding to reckless laparoscopic explorations. Conservative measures, including bowel decompression, should be considered as the primary aim of treatment in patients with Chilaiditi syndrome.

## References

[1] Yin AX, Park GH, Garnett GM, Balfour JF (2012). Chilaiditi syndrome precipitated by colonoscopy: a case report and review of the literature. Hawaii J Med Public Health.

[2] Kang D, Pan AS, Lopez MA, Buicko JL, Lopez-Viego M (2013). Acute abdominal pain secondary to Chilaiditi syndrome. Case Rep Surg.

[3] Dsouza S, Mhaske Y, Kulkarni A, Baviskar A (2018). Chilaiditi syndrome—a clinical conundrum!. South Afr J Anaesth Analg.

[4] de Acosta Andino DA, Aberle CM, Ragauskaite L, Khair G, Streicher A, Bartholomew J, Kacey D (2012). Chilaiditi syndrome complicated by a closed-loop small bowel obstruction. Gastroenterol Hepatol.

[5] Gad MM, Al-Husseini MJ, Salahia S, Saad AM, Amin R (2018). Chilaiditi syndrome-a rare case of pneumoperitoneum in the emergency department: a case report. J Med Case Rep.

[6] Lin CH, Yu JC, Ou JJ, Lee YT, Huang M, Wu HS (2012). Chilaiditi syndrome: the pitfalls of diagnosis. Surg Sci.

[7] Kapania EM, Link C, Eberhardt JM (2018). Chilaiditi syndrome: a case report highlighting the intermittent nature of the disease. Case Rep Med.

[8] Gurvits GE, Lau N, Gualtieri N, Robilotti JG (2009). Air under the right diaphragm: colonoscopy in the setting of Chilaiditi syndrome. Gastrointest Endosc.

